# The Association Between COVID-19 and Herpes Zoster in Adult Populations: A Systematic Review

**DOI:** 10.7759/cureus.112344

**Published:** 2026-07-09

**Authors:** Iqra Khawaja, Nauman Khan, Lushanthi Kannangara, Sumedha N Panangala Liyanage, Ethel Gomez Canales, Sarvjit S Sahedra

**Affiliations:** 1 Internal Medicine, Northampton General Hospital NHS Trust, Northampton, GBR; 2 Geriatric Medicine, Northampton General Hospital NHS Trust, Northampton, GBR; 3 Geriatrics, Northampton General Hospital NHS Trust, Northampton, GBR; 4 Geriatric Medicine, Great Western Hospitals NHS Foundation Trust, Swindon, GBR; 5 General Internal Medicine, University Hospitals of Leicester NHS Trust, Leicester, GBR

**Keywords:** covid-19, sars cov-2, shingles, skin rash, herpes zoster

## Abstract

Coronavirus disease 2019 (COVID-19), caused by severe acute respiratory syndrome coronavirus 2 (SARS‑CoV‑2), has been associated with a wide range of dermatological manifestations. Herpes zoster (HZ), also known as shingles, has been increasingly reported in adults with COVID‑19 infection. Several reports suggest that SARS‑CoV‑2-associated immune dysregulation, particularly lymphopenia and impaired T-cell-mediated immunity, may contribute to varicella-zoster virus (VZV) reactivation. This systematic review aimed to evaluate the temporal relationship between COVID‑19 infection and shingles rash, including the onset, clinical outcomes, prognostic implications, recovery, and relationship with lymphopenia. This study was performed following the Preferred Reporting Items for Systematic Reviews and Meta-Analyses (PRISMA) guidelines. A systematic literature review was conducted using PubMed, MEDLINE and Cochrane databases for studies published within the last five years. Search terms included “COVID‑19”, “SARS-CoV-2”, “herpes zoster” and “shingles rash”. Inclusion criteria consisted of case reports, case series, cohort studies, systematic reviews, and research studies. Studies included were in the English language. Studies not relevant to the research question, paediatric cohorts, or commenting on vaccination were excluded. Fifty-seven studies (N=57) were initially identified through database search, with 35 studies (N=35) deemed relevant to the research question following screening. Twenty-five studies (N=25) were excluded due to data not being relevant to the research question, paediatric cohort data, or vaccination-related data. After exclusion, 10 studies (N=10) were included in the review for qualitative analysis. Most studies demonstrated a temporal association between COVID‑19 infection and HZ occurrence. HZ rash developed from two days before COVID‑19 symptoms and up to 70 days after infection, with an average onset approximately 17 days after COVID‑19 diagnosis. Several studies reported that HZ preceded or coincided with COVID‑19 symptoms, suggesting that shingles may serve as an early indicator of SARS‑CoV‑2 infection. Associated lymphopenia was noted, suggesting that COVID-19-induced immune dysregulation contributes to VZV reactivation. Most patients recovered from HZ rash following antiviral therapy, although severe complications and prolonged hospitalisation were observed in cases involving co-infection and systemic disease. Overlooking this possible association may result in an undiagnosed COVID-19 infection in those presenting with shingles rash and a higher risk of developing long COVID. The reviewed studies indicate that COVID-19 may be associated with HZ reactivation in the general adult population, although causality remains unproven. This detailed data analysis and systematic review demonstrate that evidence is scarce in this domain, which warrants larger epidemiological and immunological studies as well as further meta-analyses to determine the prognostic significance of shingles rash in COVID‑19 infection, in particular long COVID.

## Introduction and background

Coronaviruses are single-stranded RNA viruses belonging to the family Coronaviridae. Three highly pathogenic coronaviruses have emerged affecting humans, resulting in significant global health concerns. Severe acute respiratory syndrome coronavirus (SARS-CoV-1), first identified in China in 2002, caused an outbreak of severe respiratory illness with a mortality rate of approximately 10% [1]. Middle East respiratory syndrome coronavirus (MERS-CoV), first reported in Saudi Arabia in 2012, was associated with outbreaks of severe respiratory disease and a higher mortality rate of approximately 34% [2]. In late 2019, severe acute respiratory syndrome coronavirus 2 (SARS-CoV-2) emerged in Wuhan, China, causing coronavirus disease 2019 (COVID-19), which spread rapidly worldwide [3].

While most individuals recover from infection, there have been reports of individuals with persistent or recurrent symptoms lasting weeks to months after the initial illness, a condition commonly referred to as long COVID or post-COVID-19 syndrome. Long COVID may involve fatigue, dyspnoea, cognitive impairment, neurological symptoms, cardiovascular manifestations, and a range of other complications [4]. The morbidity of COVID-19 disease has been overlooked, with patients diagnosed with long-COVID syndrome experiencing multisystemic manifestations for over seven months, causing a significant impact on their quality of life [5]. COVID‑19 rapidly evolved into a global pandemic following the emergence of SARS‑CoV‑2 in late 2019. Although primarily considered a respiratory illness, COVID‑19 has been associated with numerous extrapulmonary manifestations, including neurological, cardiovascular, gastrointestinal, and dermatological complications [6].

Cutaneous manifestations in COVID‑19 were investigated in a nationwide study conducted in Spain, which reported maculopapular eruptions (47%), chilblain-like acral lesions (19%), urticarial lesions (19%), vesicular eruptions (9%), and livedoid or necrotic lesions (6%) [7]. Among these dermatological findings, herpes zoster (HZ) has increasingly attracted attention due to reports suggesting a potential association with SARS‑CoV‑2 infection [8].

Varicella-zoster virus (VZV) causes chickenpox in children and shingles rash in adults. Shingles rash and HZ rash are interchangeable terms. HZ results from reactivation of latent VZV within sensory dorsal root ganglia. Reactivation is commonly associated with advancing age, immunosuppression, malignancy, stress, and impaired cellular immunity [9]. SARS‑CoV‑2 infection has been shown to produce immune dysregulation characterised by lymphopenia, T-cell exhaustion, cytokine imbalance, and impaired interferon responses [10,11]. These immunological changes may predispose infected individuals to reactivation of latent herpes viruses, including VZV [12].

During the COVID‑19 pandemic, there have been reports describing increased rates of HZ diagnosis as well as cases in which shingles preceded, coincided with, or followed COVID‑19 infection. Some authors have proposed that HZ may serve as an early clinical indicator of underlying COVID‑19 infection, while others suggest that COVID‑19-related immune dysfunction may directly contribute to VZV reactivation [8,13]. There have also been studies reporting a higher incidence of HZ in patients with confirmed COVID-19 infection compared with uninfected controls [14].

Despite increasing reports of COVID‑19-associated HZ, no comprehensive systematic review or meta-analysis has yet established a definitive causal relationship. Therefore, this systematic review aimed to evaluate the current evidence regarding the association between COVID-19 and HZ, including the timing of rash onset, clinical outcomes, prognostic implications, and the potential role of lymphopenia in VZV reactivation.

## Review

Methods

Study Design and Reporting Framework

A systematic literature review was conducted in accordance with the Preferred Reporting Items for Systematic Reviews and Meta-Analyses (PRISMA) guidelines [15]. The review process adhered to established methodological standards for diagnostic accuracy studies. The objective was to evaluate the temporal relationship between COVID‑19 infection and shingles rash, including the timing of onset, clinical outcomes, prognostic implications, recovery, and the association with lymphopenia.

Eligibility Criteria

Studies were considered eligible for inclusion if they were published within the previous five years and relevant to the research question. Eligible study designs included case reports, case series, cohort studies, systematic reviews, and other original research studies. Studies were required to involve adult patients with confirmed COVID-19 infection and concurrent HZ or VZV reactivation. Articles were screened for relevance to the research question, and studies that provided clinical information on the association between COVID-19 and HZ were included.

Studies were excluded if they involved vaccine-related data, paediatric populations or did not provide sufficient clinical data relevant to the study objectives. Non-English language publications were excluded due to limitations in data extraction and interpretation. Studies that did not investigate HZ or VZV reactivation in the context of COVID-19 infection were also excluded from the review.

Information Sources and Study Selection

**Figure 1:**
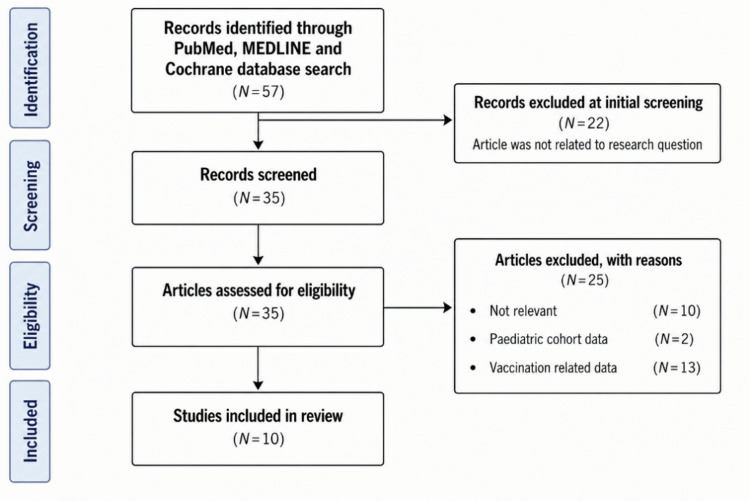
PRISMA flow diagram of the selection of studies PRISMA: Preferred Reporting Items for Systematic Reviews and Meta-Analyses

PubMed, MEDLINE and Cochrane databases were searched for studies published within the previous five years. Search terms included “COVID‑19”, “SARS-CoV-2”, “herpes zoster” and “shingles rash”. Fifty-seven studies (N=57) were initially identified through database search, with thirty-five studies (N=35) deemed relevant to the research question following screening. Twenty-five studies (N=25) were excluded due to unrelated data, paediatric cohort data, or vaccination-related data. After exclusion, 10 studies (N=10) were included in the review for qualitative analysis. Figure 1 shows the PRISMA flow diagram of the selection of studies.

Data Extraction

Data were extracted from all included studies. Information collected included study characteristics such as study design, publication year, and patient demographics. Clinical variables extracted included the timing of HZ onset relative to COVID-19 infection, the distribution and severity of the rash, the presence of lymphopenia, the severity of COVID-19 disease, antiviral treatments administered and reported clinical outcomes. Outcome measures included recovery, mortality, morbidity, and any reported prognostic implications of HZ in patients with COVID-19.

Subsequently, the extracted data were synthesised to evaluate the potential association between SARS-CoV-2 infection and VZV reactivation, as well as the role of HZ as a possible clinical indicator of disease severity.

Quality Assessment and Risk of Bias

The quality of the included studies was independently assessed by two investigators (IK and NK) according to study design. Any disagreements regarding the risk of bias were resolved by a third author (LK). Retrospective cohort studies were evaluated using the ROBINS-I tool [16]. Systematic reviews were assessed using the AMSTAR 2 checklist [17]. Case reports were appraised using the Joanna Briggs Institute (JBI) Critical Appraisal Checklist for Case Reports [18].

Data Synthesis and Analysis

Continuous variables were displayed as means and medians (with interquartile ranges), whereas categorical variables were presented as counts (with percentages).

Ethical Considerations

As this study did not contain any new studies with human participants, ethics committee approval was not required. The data and conclusions drawn in this systematic review were based on previously conducted studies and case reports.

Study Selection Process

The final review included 10 studies comprising three case reports and case series, three retrospective cohort studies, and four systematic reviews, one of which also described a case report. The studies collectively evaluated 9,469,648 patients with either confirmed SARS‑CoV‑2 infection and concurrent HZ or populations at increased risk for HZ following COVID‑19 infection. The included studies demonstrated considerable heterogeneity in design, patient population, and reported outcomes.

Characteristics of Included Studies

Table 1 summarises the characteristics of the studies included in this review. Three case reports, as well as a systematic review that included an original case report, describing four individual cases of HZ were included in the analysis of the systematic review. Data including age (years), sex, comorbidities, distribution of rash, onset of rash in relation to COVID-19 infection, duration of rash, treatment, evolution and lymphocyte count were extracted. Patients ranged in age from 25 to 62 years, with two male patients and two female patients. Two patients presented with localised truncal rashes, while two developed disseminated disease. The timing of onset varied, occurring one week and three days before COVID-19 diagnosis in two patients and four days after COVID-19 diagnosis in one patient. Antiviral therapy was the mainstay of treatment, including oral or intravenous acyclovir. Outcomes were generally favourable, with two uncomplicated recoveries. However, one patient developed severe COVID-19, disseminated VZV infection, and polymicrobial tracheobronchitis requiring intensive care admission and prolonged hospitalisation. Lymphocyte counts were reported in two cases and demonstrated lymphopaenia, including one patient with a lymphocyte count of 0.5 × 10⁹/L. Overall, these cases demonstrated heterogeneity in rash distribution and timing but highlighted the occurrence of HZ both before and after COVID-19 infection, with lymphopaenia present where data were available.

**Table 1:** Characteristics of the included studies. HZ, herpes zoster; VZV, varicella-zoster virus; COVID-19, coronavirus disease 2019; SARS-CoV-2, severe acute respiratory syndrome coronavirus 2; IBD, inflammatory bowel disease; aOR, adjusted odds ratio; RR, relative risk; CI, confidence interval; IRR, incidence rate ratio.

Author	Year	Study Design	Population	Outcome Measures	Key Findings
Algaadi [8]	2021	Systematic review	29 patients with HZ associated with COVID-19 infection	Timing of HZ rash, clinical distribution, treatment, lymphocyte count, and possible association with COVID-19	HZ occurred in temporal association with COVID-19; the mean patient age was 56 years; lymphopenia was reported in 86.6% of patients.
Czech and Nishimura [19]	2022	Systematic scoping review	19 articles; 25 patients with primary VZV infection or VZV reactivation in the context of COVID-19	Clinical characteristics, timing of VZV rash, distribution, dissemination, and lymphocyte count	Median time from respiratory symptom onset to VZV-related rash was 7 days; 48% had disseminated VZV infection; trunk involvement and trigeminal involvement were commonly reported.
Martinez-Reviejo et al. [20]	2022	Systematic review	55 included articles overall; 39 VZV events in patients with COVID-19	VZV reactivation or infection after COVID-19: latency, severity, complications, and clinical outcomes	The median latency was approximately 7 days; uncomplicated dermatomal HZ was the most frequent presentation; severe cases included HZ ophthalmicus.
Thada et al. [21]	2022	Case report and systematic review	79 patients from the systematic review, plus one additional case report	Timing of HZ relative to COVID-19, clinical features, treatment, morbidity, mortality, leukopenia, and lymphopenia	The mean interval from COVID-19 symptom onset to HZ was approximately 15.5 days, with a median of 7 days; some patients developed HZ before COVID-19 symptoms; mortality was not reported among the included HZ/COVID-19 cases.
Desai et al. [14]	2024	Retrospective propensity-matched cohort study	7,912 adults with IBD and SARS-CoV-2 infection in the pre-vaccine era; matched comparison cohort after propensity matching	Risk of HZ after SARS-CoV-2 infection among adults with IBD; risk of HZ-related complications	IBD patients with SARS-CoV-2 infection had increased HZ risk compared with IBD controls in the pre-vaccine era (aOR 2.16, 95% CI 1.53-3.04). No significant increase was found for HZ complications (aOR 0.87, 95% CI 0.44-1.75).
Correcher-Martinez et al. [22]	2024	Population-based retrospective cohort study	4,085,590 adults in Spain, aged ≥18 years	Incident HZ risk among adults with laboratory-confirmed SARS-CoV-2 infection compared with SARS-CoV-2-free individuals	Adults with SARS-CoV-2 infection had higher HZ risk than adults without SARS-CoV-2 (adjusted RR 1.19, 95% CI 1.09-1.29). Among adults aged ≥50 years, adjusted RR was 1.16 (95% CI 1.05-1.29).
Maldonado et al. [13]	2021	Case series	Three herpesvirus cases: two VZV infections and one HSV-1 infection; SARS-CoV-2 infection was confirmed in one of the three cases	Herpesvirus reactivation during the COVID-19 pandemic; clinical distribution and evolution	Reported herpes virus lesions during the COVID-19 pandemic, including two VZV cases; the paper discusses pandemic-related immune and stress mechanisms, but confirmed SARS-CoV-2 infection was limited.
Bruno et al. [23]	2022	Case report	One immunocompetent adult male with SARS-CoV-2 infection, primary VZV infection, and polymicrobial ventilator-associated tracheobronchitis	Severe coinfection, systemic morbidity, intensive care course, and hospital outcome	The patient developed severe multi-pathogen illness requiring intensive care and mechanical ventilation; total hospitalisation was 46 days before discharge to rehabilitation.
Tomoki Mizuno et al. [24]	2025	Self-controlled case series (retrospective claims database study)	399,381 patients with COVID-19; among these, 558 were diagnosed with shingles	Relationship between shingles and COVID-19	The IRR was significantly elevated during the first and second (5.1, 95% CI 3.9-6.6), third and fourth (1.7, 95% CI 1.2-2.5), and fifth and sixth weeks (1.5, 95% CI 1.0-2.3) compared with a control period.
Kim [25]	2024	Nationwide population-based matched cohort study	4,976,589 COVID-19 patients	Rate of herpes zoster in COVID-19 infection	Chronic urticaria, vitiligo, alopecia areata, and herpes zoster manifested at higher rates within the COVID-19 cohort.

Three retrospective cohort studies were analysed in this systematic review [14,22,25]. They provided invaluable data on the risk of HZ in adults with confirmed COVID-19 infection. One of the studies analysed 7,912 patients with inflammatory bowel disease and found that SARS-CoV-2 infection was associated with more than double the risk of HZ compared with matched controls without COVID-19 (aOR 2.16, 95% CI 1.53-3.04) [14]. There was no significant increase in HZ-related complications between the groups, whereas the larger cohort study included 4,085,590 adults and reported an overall HZ incidence of 5.76 cases per 1,000 person-years [22]. Individuals with confirmed COVID-19 had a 19% higher risk of developing HZ (adjusted RR 1.19, 95% CI 1.09-1.29), while adults aged ≥50 years had a 16% higher risk (adjusted RR 1.16, 95% CI 1.05-1.29) [22]. Furthermore, hospitalised COVID-19 patients demonstrated a greater risk of HZ than non-hospitalised patients [22]. This data demonstrated an increased risk of HZ following COVID-19 infection, thus supporting a potential association between COVID-19 and VZV reactivation.

Four systematic reviews comprising between 25 and 79 patients were included for review [8,19-21]. Across these studies, the age of patients ranged from 54 to 62 years, with a slight female predominance reported in four reviews [8,19-21]. Hypertension and diabetes mellitus were the most reported comorbidities. The clinical presentation of HZ varied. Although dermatomal eruptions were most common. Trigeminal involvement was reported in 41.3% of patients in the study by Algaadi [8]. Martinez-Reviejo et al. reported seven cases of HZ ophthalmicus and 17.1% ophthalmic involvement [20], while Czech and Nishimura also described frequent trigeminal involvement [19]. Disseminated disease was observed in up to 48% of patients [19]. The timing of HZ onset ranged from two days before to 70 days after COVID-19 symptoms, with an average onset of approximately 15-17 days after COVID-19 diagnosis [8,21]. Approximately 10-19% of patients developed HZ before or concurrently with COVID-19 symptoms [8,21]. Lymphopaenia was reported in 86.6% of patients by Algaadi [8] and in 31.6% by Thada et al. [21]. Most patients received antiviral therapy and experienced favourable outcomes. Thada et al. reported that 52.6% of patients required hospitalisation and 7.5% required mechanical ventilation [21]. No mortality directly attributable to HZ was reported across the included reviews. The systematic reviews demonstrated a temporal association between COVID-19 infection and HZ reactivation, with lymphopenia frequently observed among affected patients.

Quality Assessment

The quality assessment of the included studies is summarised in Table 2. Risk of bias was assessed according to the study design. Systematic reviews were assessed using AMSTAR-2, retrospective cohort studies were assessed using ROBINS-I, and case reports were assessed using the JBI critical appraisal checklist for case reports [16-18]. As this systematic review included evidence that consisted of heterogeneous study designs, a single appraisal tool was not applied to all studies.

The two retrospective cohort studies were judged to have a moderate risk of bias [14,22], mainly due to the observational design, possible residual confounding, and potential misclassification of outcome in healthcare databases. The systematic reviews and scoping review were judged to have a moderate risk of bias because most evidence came from case reports or case series, with differences in diagnostic confirmation, timing of rash onset, laboratory reporting, and clinical outcome reporting. The case reports were considered high risk of bias because they involved small numbers of patients, lacked comparative data, and were vulnerable to confounding. Overall, the available evidence supports a possible temporal and epidemiological association between COVID-19 and HZ reactivation rather than proof of causality.

**Table 2:** Risk of bias assessment of included studies. HZ, herpes zoster; VZV, varicella-zoster virus; COVID-19, coronavirus disease 2019; SARS-CoV-2, severe acute respiratory syndrome coronavirus 2; JBI, Joanna Briggs Institute.

Study	Study design	Tool used	Key domains considered	Main concerns identified	Overall risk of bias	Applicability concern
Algaadi (2021) [8]	Systematic review	AMSTAR-2	Search strategy; study selection; data extraction; assessment of included studies; synthesis	No clear registered protocol identified; evidence mainly from case reports; limited formal appraisal of primary studies; heterogeneity prevented quantitative synthesis.	Moderate to high concern	Useful for timing and lymphopenia signals, but causal inference remains limited.
Czech and Nishimura (2022) [19]	Systematic scoping review	AMSTAR-2	Search strategy; inclusion criteria; evidence mapping; transparency of synthesis	Scoping-review design appropriately maps evidence but does not provide pooled estimates; included evidence largely case reports; risk-of-bias appraisal of primary studies was limited.	Moderate concern	Useful for clinical pattern mapping but not designed to establish causality.
Martinez-Reviejo et al. (2022) [20]	Systematic review	AMSTAR-2	Eligibility criteria; subgroup relevance; synthesis; handling of heterogeneity	Review includes both vaccination-related and infection-related VZV events; only the COVID-19 infection subgroup is relevant to this manuscript; primary evidence is heterogeneous.	Moderate concern	Use only infection-related subgroup data; avoided generalising vaccination data to infection-related HZ.
Thada et al. (2022) [21]	Systematic review and case report	AMSTAR-2 plus JBI case-report considerations	Search and selection; data extraction; case-report reporting; outcome reporting	Mixed design combining one case report with a systematic review; evidence is largely descriptive; heterogeneity and inconsistent laboratory reporting limit pooled interpretation.	Moderate concern	Helpful for timing, morbidity, and outcomes; interpreted prognostic claims cautiously.
Desai et al. (2024) [14]	Retrospective propensity-matched cohort study	ROBINS-I	Confounding; participant selection; exposure classification; outcome ascertainment; missing data; reporting	Propensity matching strengthens comparability, but residual confounding, database coding errors, and healthcare-testing bias remain possible.	Moderate risk of bias	Provides stronger epidemiological evidence of association, but observational design prevents causal confirmation.
Correcher-Martinez et al. (2024) [22]	Population-based retrospective cohort study	ROBINS-I	Confounding; exposure classification; outcome measurement; missing data; reporting	Large database and adjustment for comorbidities strengthen the study; residual confounding and misclassification of SARS-CoV-2 or HZ diagnoses remain possible.	Moderate risk of bias	Strongest population-level evidence, but still observational and not definitive for causality.
Maldonado et al. (2021) [13]	Case series	JBI critical appraisal checklist for case reports	Patient description; diagnostic ascertainment; clinical course; outcomes; causality	Small number of cases; not all herpes virus cases had confirmed SARS-CoV-2 infection; no comparator group; causality cannot be inferred.	High risk of bias	Use cautiously as descriptive evidence only.
Bruno et al. (2022) [23]	Case report	JBI critical appraisal checklist for case reports	Patient history; diagnostic confirmation; intervention; clinical course; outcome	Single severe case with multi-pathogen coinfection; primary VZV coinfection rather than typical HZ reactivation; no comparator group; multiple confounders for morbidity.	High risk of bias	Useful for showing severe coinfection morbidity, not for estimating HZ risk after COVID-19.
Mizuno et al. [24]	Self-controlled case series (retrospective claims database study)	ROBINS-I	Confounding; participant selection; exposure classification; outcome measurement; missing data; outcome reporting	Self-controlled design reduces time-invariant confounding; however, reliance on administrative claims data may result in exposure and outcome misclassification, limited adjustment for time-varying confounders, and potential diagnostic or healthcare-seeking bias following COVID-19.	Low to moderate risk of bias	Highly applicable for assessing the temporal association between COVID-19 infection and herpes zoster in routine clinical practice; findings should be interpreted cautiously because claims databases cannot establish biological causality or capture all clinical confounders.
Kim [25]	Nationwide population-based matched cohort study	ROBINS-I	Confounding; participant selection; exposure classification; outcome measurement; missing data; follow-up completeness	Large population-based cohort and matched comparison reduce selection bias; residual confounding, dependence on routinely collected diagnostic codes, surveillance bias after COVID-19 diagnosis, and lack of randomization remain important limitations.	Moderate risk of bias	Highly applicable for estimating the population-level incidence of herpes zoster after COVID-19 infection; results support epidemiological associations but should not be interpreted as evidence of causation.

Discussion

Correlation Between COVID‑19 Infection and Herpes Zoster

This systematic review identified increasing evidence of HZ reactivation in COVID-19 infection, but an association could not be established as there is no sufficient data to infer that SARS-CoV-2 infection results in VZV reactivation. Although the currently available evidence is predominantly case reports, retrospective cohort studies, and systematic reviews, multiple publications have consistently demonstrated a temporal relationship between SARS-CoV-2 infection and the development of a shingles rash. This includes two large retrospective cohort studies, conducted by Desai et al. and Correcher-Martínez et al., which have shown a significantly increased risk of HZ among patients with COVID-19 compared with non-infected controls, providing epidemiological evidence for a potential association [14,22].

This association can be further supported by the immunopathogenesis of both COVID-19 and VZV reactivation. Following primary infection, VZV is latent within the sensory dorsal root and cranial nerve ganglia. This immunopathogenesis has also been described by Cohen in 2013 [9]. Maintenance of latency depends on intact immunity responses, particularly CD4+ and CD8+, B-cell lymphocytes, natural killer (NK) cells and total lymphocyte count. This means reactivation occurs when cellular immunity declines. Huang et al. discussed the same mechanism in a meta-analysis [10]. This reactivation results in viral replication and migration of the virus along sensory nerves to the skin, producing the characteristic dermatomal rash. SARS-CoV-2 infection has been shown to induce profound immune dysregulation in NK cells and CD8 T-cells, as discussed by Zheng et al. in 2020 [11]. The reduction in immunological response may reduce the host’s ability to control the latent herpes viruses and provide an opportunity for VZV reactivation during or following COVID-19 infection.

A larger retrospective cohort study was conducted in 2024 by Correcher-Martínez et al. to evaluate the risk of HZ in adults with COVID-19 infection. The study included 4,085,590 individuals in Spain and reported a 19% increased risk of HZ among individuals with confirmed SARS-CoV-2 infection compared with non-infected controls [22]. Furthermore, Algaadi’s study, which was conducted in Brazil, also discussed the increased incidence of HZ with COVID-19 infection, with an overall increase of 35.4% [8]. Desai et al. demonstrated that patients with inflammatory bowel disease and SARS-CoV-2 infection had more than twice the risk of developing HZ compared with matched controls (adjusted odds ratio 2.16, 95% confidence interval 1.53-3.04), with the greatest risk occurring within the first 90 days after infection [14]. Collectively, these findings support an association between COVID‑19 infection and VZV reactivation.

Timing of Herpes Zoster Rash Relative to COVID‑19 Infection

Moreover, an important observation was the variable timing of HZ onset relative to COVID-19 symptoms. In some patients, shingles developed after the onset of respiratory symptoms, whereas in others, HZ either preceded or occurred concurrently with COVID-19 manifestations. Algaadi reported that HZ developed from two days before the onset of COVID-19 symptoms to 70 days after infection, with an average onset of 17 days following COVID-19 diagnosis [8]. Similarly, Thada et al. reported a mean interval of 15.49 days (median: 7 days, n = 43) between COVID-19 symptom onset and HZ rash development, although 19% of patients developed HZ before the onset of respiratory symptoms [21]. Czech et al. additionally reported a median latency period of seven days between respiratory symptom onset and VZV-related rash development [19]. These findings suggest that HZ may occur throughout the course of SARS-CoV-2 infection and may precede classical respiratory manifestations in some cases. Maldonado et al. described a 25-year-old woman who initially presented with vesicular rash on her right lumbar area, which later spread to her arms and legs. She was initially treated for VZV Infection. However, she continued to deteriorate and started having respiratory symptoms in the subsequent seven days and tested positive for COVID-19 infection [13]. These observations suggest that HZ may occasionally serve as an early indicator of underlying SARS-CoV-2 infection, particularly in asymptomatic or minimally symptomatic patients. Therefore, clinicians should maintain a high index of suspicion for COVID-19 in patients presenting with acute shingles during periods of increased viral transmission, as HZ may occasionally serve as an early clinical indicator of COVID‑19 infection.

Clinical Presentation and Rash Characteristics

Studies also reported that HZ cases involved localised dermatomal vesicular eruptions. In the systematic review conducted by Correcher-Martínez et al., uncomplicated dermatomal HZ accounted for 68.3% of reported cases [22]. Czech et al. reported that thoracic and truncal regions were the most frequently affected anatomical sites, with truncal involvement in 60% of patients. Although most patients developed localised dermatomal disease, atypical and disseminated presentations were also observed. Czech et al. reported disseminated VZV infection in 48% of included patients. In addition, necrotic lesions were reported in 16% of cases [19].

The reviewed studies also demonstrated notable patterns in rash distribution. Algaadi reported trigeminal nerve involvement in 41.3% of patients, with 24.1% of cases involving HZ ophthalmicus [8]. Similar findings were reported by Czech et al., who observed trigeminal involvement in approximately 40% of cases [19]. Another study by Martinez-Reviejo et al. reported 17.1% cases of HZ ophthalmicus without vaccination [20]. These observations are clinically significant because ophthalmic involvement may lead to severe ocular complications, including keratitis, uveitis, and visual impairment if treatment is delayed. Prompt diagnosis and initiation of antiviral therapy are therefore essential in these patients.

Relationship Between COVID‑19 and Lymphopenia

One of the most significant findings across the included studies was the frequent presence of lymphopenia in patients with COVID-19-associated HZ. Algaadi reported lymphopenia in 13 of 15 patients (86.6%) for whom lymphocyte counts were available [8]. Likewise, Czech et al. reported a median lymphocyte count of 0.67 × 10³/μL among patients with COVID-19-associated VZV infection [19]. These findings are clinically relevant because cell-mediated immunity, particularly CD4+ and CD8+ T lymphocyte activity, plays a critical role in maintaining VZV latency as discussed above in immunopathogenesis.

Prognosis, Recovery, Morbidity, and Mortality

The prognostic significance of HZ in COVID-19 remains uncertain. Some authors proposed that shingles may reflect underlying immune dysfunction and potentially correlate with more severe COVID-19 disease. However, current evidence is insufficient to establish HZ as a definitive prognostic marker for morbidity or mortality. Further systematic reviews, meta-analyses and larger prospective studies are needed to evaluate whether shingles occurrence correlates with disease severity or long-term outcomes in COVID-19 patients.

Most patients included in the reviewed studies recovered following standard antiviral treatment with acyclovir, valacyclovir, or famciclovir [8,21]. Intravenous acyclovir was generally reserved for severe or disseminated disease. Although overall outcomes were favourable, significant morbidity was observed in patients with severe systemic involvement. Bruno et al. described a patient with concurrent SARS-CoV-2 infection, primary VZV infection, and polymicrobial ventilator-associated tracheobronchitis who required intensive care admission, mechanical ventilation, and a prolonged hospital stay of 46 days before transfer to rehabilitation [23]. While mortality directly attributable to HZ was not consistently reported, these findings suggest that coexisting viral infections may contribute to worse clinical outcomes and prolonged hospitalisation. Mortality specifically attributable to HZ in COVID‑19 patients was not consistently reported across all studies.

Strengths and Limitations

The number of available studies evaluated in this review remains limited, with most evidence derived from case reports and retrospective observational studies. This limits the ability to establish causality. Furthermore, the heterogeneity in study design, patient populations, and outcome reporting prevented formal meta-analysis. Several confounding factors, such as advanced age, stress, immunosuppressive therapy, and comorbid conditions, may independently contribute to VZV reactivation. Additionally, there was inconsistent reporting of laboratory findings and long-term follow-up data, which limited evaluation of prognosis and recurrence risk.

Despite these limitations, the available evidence suggests a likely association between COVID-19 infection and HZ reactivation. SARS-CoV-2-associated immune dysregulation, particularly lymphopaenia and impaired cellular immunity, appears to play a central role in this process. This detailed data analysis and systematic review demonstrate that evidence is scarce in this domain, which warrants larger epidemiological and immunological studies as well as further meta-analyses to determine the prognostic significance of shingles rash in COVID‑19 infection.

## Conclusions

Current evidence suggests a possible association between COVID‑19 infection and HZ reactivation. SARS‑CoV‑2-related immune dysregulation, particularly lymphopenia and impaired cellular immunity, may facilitate VZV reactivation. Furthermore, HZ may occasionally precede or coincide with classical COVID‑19 symptoms and could therefore represent an early indicator of infection in some patients. Most patients recover following antiviral therapy; however, severe morbidity may occur in cases involving disseminated disease or systemic co-infection. Overlooking this possible association may result in an undiagnosed COVID-19 infection in those presenting with shingles rash and a higher risk of developing long COVID, with implications later. Despite increasing evidence, the currently available data remain insufficient to establish definitive causality. Surprisingly, there has been no meta-analysis on this domain due to a lack of reported evidence. Thus, larger epidemiological studies and meta-analyses are required to clarify the relationship between COVID‑19 and shingles and to determine the prognostic significance of HZ in SARS‑CoV‑2 infection, as there is a lack of adequate experimental studies elucidating the impact of SARS-CoV-2 and VZV co-infection.
